# Development of a Dynamic Path Planning System for Autonomous Mobile Robots Using a Multi-Agent System Approach

**DOI:** 10.3390/s25175317

**Published:** 2025-08-27

**Authors:** Bradley Fourie, Louis Louw, Günter Bitsch

**Affiliations:** 1 Department of Industrial Engineering, Stellenbosch University, Stellenbosch 7600, South Africa; 2 NXT Nachhaltigkeit und Technologie, Reutlingen University, 72762 Reutlingen, Germany; guenter.bitsch@reutlingen-university.de

**Keywords:** Autonomous Mobile Robots (AMRs), dynamic path planning, Multi-Agent Systems (MAS), Flexible Manufacturing Systems (FMS)

## Abstract

Autonomous Mobile Robots (AMRs) are increasingly important in Industry 4.0 intralogistics but creating path planning systems that adapt to dynamic and uncertain Flexible Manufacturing Systems (FMS), especially managing conflicts among multiple AMRs with a need for scalable decentralised solutions, remains a significant challenge. This research introduces a dynamic path planning system for AMRs designed for reactive adaptation to FMS disturbances and generalisation across factory layouts, incorporating support for multiple AMRs with integrated conflict avoidance. The system is built on a Multi-Agent Systems (MAS) architecture, where software AMR agents independently calculate their paths using a hybrid Genetic Algorithm (GA) that employs Cell-Based Decomposition (CBD) and optimises path length, smoothness, and overlap via a multi-objective fitness function. Multi-AMR conflict avoidance is implemented using the Iterative Exclusion Principle (IEP), which facilitates priority-based planning, knowledge sharing through Predictive Collision Avoidance (PCA), and iterative replanning among agents communicating via a blackboard agent. Verification demonstrated the system’s ability to successfully avoid deadlocks for up to nine AMRs and exhibit good scalability. Validation in a simulated FMS environment confirmed robust adaptation to various disturbances, including static and dynamic obstacles, while maintaining stable run times and consistent path quality. These results affirm the practical feasibility of this hybrid GA and MAS-based approach for dynamic AMR control in complex industrial settings.

## 1. Introduction

In the context of Industry 4.0 and the evolution of smart autonomous systems, Autonomous Mobile Robots (AMRs) are attracting significant interest across various industrial applications [1,2]. Specifically, AMRs have demonstrated effectiveness in intralogistics tasks such as material handling within production systems, warehouses, hospitals, and intelligent container terminals [2]. These autonomously controlled robots, which include types like automated forklifts and mobile platforms, enhance manufacturing flexibility, contribute to process stability, and improve economic efficiency by minimising human errors and injuries.

However, a significant challenge in implementing autonomous AMR control systems is the development of path planning systems capable of adapting to the dynamic and uncertain environments characteristic of Flexible Manufacturing Systems (FMS) [3,4,5]. Path planning involves generating optimal paths via waypoints between specified locations for transport orders. FMS environments pose uncertainties stemming from both high-level task scheduling, such as sudden changes in transport order priorities, and low-level factory floor issues like static and dynamic obstacles, machine failures, and alterations to workstation layouts. Developing an adaptive AMR path planning system requires addressing the interrelated challenges of configuration space representation and designing an effective search algorithm. The recent literature frames path search as a multi-objective optimisation problem, incorporating factors like path length, smoothness, and safety [6].

When deploying a fleet of AMRs, conflicts such as deadlocks and collisions become inevitable. Unlike guided systems, AMRs move freely, making pre-defined traffic rules insufficient for conflict avoidance. Multi-AMR conflict avoidance focuses on adapting individual AMR paths to ensure the entire set of paths is conflict-free. While the most efficient methods involve pre-adaptation of paths during path planning, current centralised implementations of conflict avoidance systems often struggle to scale effectively to larger AMR fleets [4,7]. There remains a lack of consensus regarding the most appropriate path planning algorithms for AMRs and, crucially, on how to implement decentralised multi-AMR conflict avoidance mechanisms that scale effectively [8], which forms a fundamental rationale for this research.

Current path planning techniques for dynamic obstacles face several critical gaps that limit their effectiveness in real-world applications [4]. A major issue is the inability to perform real-time path replanning efficiently; many algorithms struggle to balance computational speed with the quality of the generated paths, especially in rapidly changing environments. Additionally, the modelling of dynamic obstacle behaviour remains inadequate, as existing probabilistic or stochastic approaches are computationally expensive and fail to fully capture complex, context-driven behaviours [9]. Scalability is another major concern, particularly in multi-robot systems, where increased numbers of agents lead to exponential growth in coordination and computation challenges, often resulting in performance degradation or system failure [10]. Finally, current methods often falter in complex environments with dense, irregular, or overlapping obstacles, and in areas with limited manoeuvrability, where finding feasible paths becomes computationally intensive and unreliable. These limitations restrict the applicability of current path-planning approaches in dynamic, unstructured, or densely populated scenarios [11].

Beyond industrial intralogistics, the complexities of dynamic path planning and conflict resolution extend to and present significant challenges in other multi-agent autonomous systems. For instance, the coordination of drone swarms requires highly efficient path planning and collision avoidance mechanisms to operate effectively in complex, often three-dimensional, dynamic environments. Research in this area, such as that highlighted in [12], demonstrate how decentralised control and inter-agent communication strategies enable collision avoidance and scalability in highly dynamic 3D environments. These principles are directly transferable to AMR fleets, where similar coordination and adaptability are needed in complex factory layouts. Furthermore, novel algorithmic approaches, including chaos-based path planning, are being explored to enhance adaptive and robust navigation in unpredictable scenarios. Works like [13] exemplify how unconventional computational paradigms can be applied to achieve resilient movement in uncertain settings. These diverse applications underscore the broad relevance and ongoing advancements in developing robust, scalable, and adaptive path planning solutions for autonomous systems across various domains.

This paper presents a dynamic path planning system for AMRs designed for reactive adaptation to disturbances in an FMS and generalisation across various factory layouts. The core purpose of this work is to extend the capabilities of dynamic path planning to support multiple AMRs through integrated multi-AMR conflict avoidance techniques, aiming to unify state-of-the-art intelligent path planning algorithms with recent advancements in multi-AMR conflict avoidance. The system utilises a Multi-Agent Systems (MAS) architecture where software AMR agents independently calculate their paths. A hybrid Genetic Algorithm (GA) formulation, employing Cell-Based Decomposition (CBD), is used for path planning, optimising path length, smoothness, and overlap. Multi-AMR conflict avoidance is implemented using the Iterative Exclusion Principle (IEP) [14], enabling priority-based planning, knowledge sharing via Predictive Collision Avoidance (PCA), and iterative replanning among communicating agents.

The main aim is to verify and validate the practical feasibility and performance of this hybrid GA and MAS-based dynamic path planning system in complex industrial scenarios. Verification experiments demonstrated the system’s ability to successfully avoid deadlocks for up to nine AMRs in a confined environment and exhibit good scalability. Validation in a simulated FMS environment confirmed robust adaptation to various disturbances, including static and dynamic obstacles and changes in transport orders, while maintaining stable run times and consistent path quality. These findings affirm the practical applicability of this approach for dynamic AMR control in challenging FMS environments.

The remainder of this paper is organised as follows: Section 2 reviews the related literature on mobile robot path planning and conflict avoidance. Section 3 details the formulation and design of the proposed path planning system and conflict avoidance mechanism. Section 4 describes the experiments verifying the system’s design and validation of the selected path planning algorithm in a simulated FMS environment. Finally, Section 5 provides the conclusions of this paper.

## 2. Related Literature

Due to the increased interest in the FMS concept, more research efforts are being directed at increasing the efficiency of the AMR path planning system to enable flexible intralogistics in these new manufacturing systems [15,16,17]. Two active fields of research exist that are paramount for the path planning of AMRs in FMSs, namely mobile robot path planning and multi-robot conflict resolution.

### 2.1. Mobile Robot Path Planning

Traditional mobile robot path planning is typically divided into static and dynamic approaches. Static path planning focuses on finding the shortest path between two nodes using global information, but it ignores dynamic obstacles and traffic conditions [6]. This makes it less suitable for modern manufacturing systems where environments are dynamic. On the other hand, dynamic path planning accounts for real-time system information and adapts to dynamic obstacles, making it more applicable to flexible manufacturing systems (FMS) [6]. Thus, path planning algorithms for autonomous mobile robots (AMRs) in FMS environments must be responsive and flexible, adapting to specific transport orders.

Early research on AMR path planning often used exact approaches. For instance, Dijkstra’s algorithm, a graph theory method, was applied to single mobile robots [18]. This research showed that Dijkstra’s algorithm could effectively handle global path planning in static environments, though the algorithm required recalculating when encountering new obstacles. Although being applied in numerous short-path planning problems, with different variations and improvements made to the algorithm, approaches using Dijkstra’s algorithm perform slowly in dynamically changing environments [5].

Despite the effectiveness of Dijkstra’s algorithm, improvements like the A∗ algorithm have become more prevalent for static path planning in AMRs [16]. The A∗ algorithm constructs a weighted environment graph and works towards the goal node by visiting neighbouring nodes, but it requires significant memory, especially for larger graphs This makes it less viable for dynamic and time-sensitive tasks [11]. Other exact approaches, like dynamic programming and dynamic time-window methods, have also been applied to AMR path planning but often struggle with problem-specific constraints and fail to perform well in dynamic, congested environments [16,17].

To address these challenges, meta-heuristic approaches have been explored for AMR dynamic path planning. Given the large solution space, exact methods can be slow to converge, posing a problem for real-time path planning. Meta-heuristic approaches like Genetic Algorithms (GAs), Particle Swarm Optimisation (PSO), and Ant Colony Optimisation (ACO) have been suggested as alternatives [15,19,20,21]. For example, a PSO implementation aimed at minimising energy consumption rather than travel distance has shown success, especially in heterogeneous AMR systems [22].

GAs have also proven effective in multi-objective AMR path planning, where objectives like minimising travel distance and avoiding obstacles are prioritised [19]. Unlike other approaches, GAs have introduced new genetic operators to improve path quality. Furthermore, comparative studies have shown that GAs tend to produce more consistent and optimal paths than PSO for complex and highly nonlinear path planning problems [23,24].

### 2.2. Mobile Robot Conflict Resolution

The integration of multiple Autonomous Mobile Robots (AMRs) within an environment introduces non-stationarity and increases the likelihood of conflicts among AMRs. Consequently, the shortest computed path may not always correspond to the fastest travel time [16]. Conflicts arise when each AMR pursues its shortest path independently, leading to system failures and significant delays. In confined spaces like Flexible Manufacturing Systems (FMSs), multiple AMRs attempting to access the same zone can cause congestion, escalating the probability of collisions and deadlocks.

AMRs can encounter various obstacle types during operation. Dynamic obstacles include other AMRs, workers moving nearby, or non-autonomous vehicles crossing planned paths. Static obstacles comprise entities like movable workstations or malfunctioning AMRs obstructing movement. Deadlocks occur when two AMRs cannot proceed along their pre-planned paths without risking collision, resulting in both remaining in a blocking state that hampers overall system efficiency [16].

Addressing these conflicts necessitates the pre-computation of conflict-free paths, especially in environments dense with static and dynamic obstacles where congestion is unavoidable [14,16,25]. This pre-emptive approach reduces the need for replanning and enhances the algorithm’s overall runtime efficiency [25]. Effective conflict resolution relies on appropriately formulating the problem to anticipate and avoid potential conflicts during path planning.

Decentralised conflict avoidance techniques in multi-robot systems typically follow a two-stage approach. First, each AMR independently computes its optimal path without considering others. Second, a coordination mechanism resolves conflicts, assigning responsibility to specific AMRs for conflict resolution [25]. The Priority-based Planning (PP) algorithm exemplifies this strategy by decomposing the complex multi-agent problem into several single-agent path planning tasks, significantly improving computational efficiency over centralised methods [25]. Although PP approaches yield sound solutions efficiently, they may not always achieve the globally optimal joint solution [25].

In [26], a PP implementation for mobile robots is presented where each robot initially plans its path individually in a dynamic environment. Robots are then assigned priorities, and conflicts are resolved sequentially based on descending priority levels, treating higher-priority robots as dynamic obstacles during resolution. This method effectively incorporates both static and dynamic obstacles and demonstrates superior scalability compared to coordinated approaches.

Further contributions to decentralised conflict avoidance include the Multi-Party Cooperative Avoidance (MPCA) algorithm proposed by [27]. This approach treats each robot as an individual agent and employs a multi-party coordinator to resolve conflicts, offering effective coordination mechanisms for collision avoidance in a decentralised framework. The MPCA algorithm has been shown to outperform previous implementations in efficiency and effectiveness [27].

Building upon these foundations, ref. [14] introduces the Iterative Exclusion Principle (IEP) for enhanced multi-AMR conflict avoidance. The IEP combines the initial path planning and priority strategies from [26] with the agent-based coordination concepts from MPCA [27]. In this framework, each mobile robot agent calculates its path and resolves its assigned conflicts while sharing knowledge with other agents, culminating in a comprehensive multi-agent architecture for effective conflict resolution in multi-robot systems [10].

## 3. Design of the Path Planning System

The FMS environment contains various static and dynamic obstacles that can obstruct the planned path of the AMRs. Due to the restricted moving space, high probability of disturbances affecting the AMRs, and time-critical nature of the delivery tasks, it is necessary that the AMRs plan their paths efficiently and in a short amount of time. The path planning algorithm applied in this study uses an updated configuration space of the environment and employs a GA search algorithm to find the most efficient path based on several metrics. Thereafter, the mobile robots have to collaboratively adapt their paths to avoid any conflicts, while prioritising the important tasks.

### 3.1. Environmental Modelling

For the environmental modelling in this study, only Cell-Based (or Grid-Based) Decomposition (CBD) methods were considered for global path planning due to its simplicity and ease of implementation [6,16,28]. The most important advantage of the CBD technique is that it is possible to automate the process by using simple computer vision techniques, as demonstrated by [29]. For the CBD approach, the environment is divided into a grid of cells, and the configuration space is constructed by identifying which cells contain obstacles and which cells contain free space [16,28]. The cells that contain full obstacles or pieces of obstacles are represented as occupied, and the robot cannot reach these states.

### 3.2. Hybrid GA Formulation

**Figure 1:**
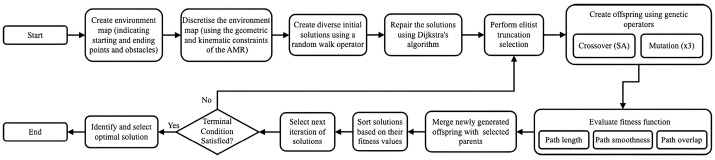
A flow diagram representation of the design of the hybrid GA for the mobile robot path planning.

The design of the GA for this work is inspired by the work performed by [19,21,30,31]. A flow diagram representing the design of the hybrid GA for the mobile robot path planning of this paper is presented in Figure 1. This approach utilises an improved initialisation function, three mutation operators, and a multi-objective fitness function. The path planning literature review performed by [15] states that GAs can successfully be combined with other algorithms to produce hybrid approaches. The hybrid GA approaches can efficiently produce valid offspring and effectively search the solution space while preserving the best solutions that have previously been found. The investigation of these hybrid GA implementations remains an active field of research in the mobile robot path planning literature, and this work aims to further contribute to this aspect.

The GA is a population-based meta-heuristic inspired by the biological phenomenon of natural evolution [32]. A starting population of solutions is initialised and through an iterative process, new solutions are generated. The new solutions are constructed using parts of the solutions from the previous generation and replacing some of the previous solutions in the population to form the new generation [32,33]. Each individual represents a solution and is encoded by a set of genes that encode the information. Each solution is evaluated using an objective function that quantifies how suitable the solution is. For the GA formulation, the objective function is called the fitness function, quantifying how well it is adapted to its environment, where fitter individuals are more likely to survive and reproduce.

For the path planning problem, the potential solutions are required to connect a starting point with an ending point through a set of neighbouring intermediary points [19,21]. These intermediary points should obey the geometric and kinematic constraints of the mobile robot and should, therefore, not include any obstacles or ragged movements. For this work, the genetic representation is designed to complement the nature of the environment, and the chromosomes are represented as a set of neighbouring coordinates that connect the starting coordinate *s* to the goal coordinate *g*. Here, each of the chromosomes contains a set of neighbouring coordinate points that the mobile robot should traverse.

Traditional methods of randomly initialising the environment produce a large amount of non-feasible solutions that, in return, slow down the convergence process. Therefore, inspiration is taken from the work performed by [21,34], and the initial population is generated using a combination of a random generation process combined with a constructive process. The collision-free paths are generated with random initial lengths through a random walk process, and these paths are then repaired by representing the environment as a directed acyclic graph and using Dijkstra’s algorithm. The corresponding population size parameter is selected based on the environment’s size to strike a balance between maintaining diversity in the solution generation process and minimising convergence time [30].

To evaluate the suitability of the individuals in the population, both the criteria associated with the efficiency of the AMR’s path and the criteria associated with the AMR’s path in relation to the other AMRs paths have to be considered. Therefore, important evaluation metrics such as path length, path smoothness, and path overlap are used to quantify the quality of the generated paths. The distance travelled is the most important consideration as it both increases the delivery time and the energy usage of the AMR [35]. Using the notation that the k’th AMR’s path is a set of coordinates denoted as $P^{k}$, $P_{1}^{k}$ is the starting coordinates of the AMR, and $P_{G}^{k}$ is the coordinates of the AMR’s goal coordinates. The total path length L of the AMR can be calculated as shown in Equation (1). Here, the ||.|| operator denotes the distance metric between the two successive coordinate sets, which can either be calculated using the Euclidean or Manhattan distance formulae.

(1) $$L\left(P^{k}\right)=\sum_{i=1}^{G-1}||P_{i+1}^{k}-P_{i}^{k}||$$

The more turns the AMR has in its trajectory, the more it is required to supply power to accelerate and decelerate its motors, resulting in a further increase in the energy usage of the AMR. Therefore, inspiration is taken from [35,36] to calculate the deflection angle between any three waypoints in the trajectory. The energy score calculation related to the smoothness of the path is given in Equation (2), which calculates the angle between the two line segments created by three waypoints.

(2) $$E\left(P^{k}\right)=\sum_{i=2}^{D-1}acos\;(\frac{\left(P_{i}^{k}-P_{i-1}^{k})(P_{i+1}^{k}-P_{i}^{k}\right)}{\left(P_{i}^{k}-P_{i-1}^{k}\right)x\left(P_{i+1}^{k}-P_{i}^{k}\right)\times 180})$$

The proposed path overlap evaluation metric is calculated using Equation (3), which evaluates the suitability of the planned path based on the fraction of the total intersection of the AMR’s path with the paths of the other AMRs. This calculation captures the idea that as the fraction of the overlap between the robot paths increases, the risk of a possible deadlock or collision in the system is also increased. The IEP algorithm from [14] is utilised, which requires that the lower-priority AMRs plan their paths after the higher-priority AMRs, and use the knowledge about the higher-priority AMR paths to plan their own paths. Therefore, the AMRs are encouraged to plan their paths in the environment’s free space or reuse their previously planned path segments instead of traversing path segments previously occupied by the other AMRs.

(3) $$S\left(P_{k}\right)=\frac{\sum_{j\neq k}^{n}L(P^{k})\cap L(P^{j})}{L(P^{k})}$$

The final fitness function design is, therefore, calculated using Equation (4). Here, weights are provided for each of the three criteria discussed above (Equations (1)–(3)) to evaluate the individuals in the population. It is important to assign the weights $\left(\omega \right)$ to each of these criteria correctly, as some of the criteria are inversely proportional. For example, adding a large weight to the path overlap score might cause paths that are not the shortest path and have significant energy scores to be chosen and vice versa.

(4) $$F\left(P_{k}\right)=\omega_{1}L\left(P^{k}\right)+\omega_{2}E\left(P^{k}\right)+\omega_{3}S(P^{k})$$

After the population has been initialised and the individuals in the population have been assigned their fitness scores, the individuals that will contribute to the next generation is selected. The selection operator is implemented in two stages to preserve the best solutions from the previous generation and is inspired by the works performed by [30,37,38]. The current population is first ordered by their fitness, and the top individuals from the current population are placed in a group to be preserved to perform the elitist and truncation selection. A roulette wheel selection operator is then employed to ensure that a diverse set of individuals reproduce and generate new solutions. The roulette wheel selection method is chosen as it has shown good results in mobile robot path planning [19,36,38,39]. A fixed population size is chosen and the selection operation results in the population being carried over having the same size as the previous generation.

The crossover operator is employed to generate new, more optimal paths by combining the genes of the two chosen parents. For this work, the genes represent the coordinate points to visit, and the swapping of genes by the crossover operator thus implies that the neighbouring coordinates to visit are then replaced and swapped between the parents to form new offspring that form part of the second group. However, the implementation of traditional crossover operators cannot be applied to the mobile robot path planning problem because randomly choosing genes to swap will lead to a large amount of non-feasible solutions being carried over to the new generation [36]. Therefore, the crossover operator needs to be carefully designed to increase the convergence rate of the modified GA.

The chosen operator for this work is based on the crossover operator employed by [30]. This operator is a domain-specific implementation of the traditional single-point crossover operator. The mechanism of this operator can be explained as follows. At the start of the crossover, both chromosomes are scanned, and a list of feasible crossover points between the two parents is generated. It is important to note that for the generation of this list, the starting and ending points of the parents are excluded, as both paths have the same starting and ending points. If the list of feasible crossover points is empty, then the two parents are deemed to be incompatible, and the selection process is repeated. However, if the list of feasible crossover points is not empty, then a random point from this list is chosen, and the single-point crossover is executed, generating two new offspring.

After the offspring are generated through the use of the crossover operator, the search space is increased by performing a random mutation on the solutions. For this work, three domain-knowledge-based operators are implemented, namely the circuit removal, the subsection replacement, and the adjacent neighbour operators. The circuit removal operator is based on the shortest operator used by [37], or the so-called circuit removal operator used by [30], or the deletion operator used by [19]. The redundant genes in the individual are removed using a list of stored repetitions, and effectively, the planned path is shortened whilst maintaining its feasibility. The subsection replacement operator further extends this idea and removes circuits that cannot be detected by seeking explicit repetitions of coordinates. The operator chooses two random genes in an individual and replans the shortest path between these two points using Dijkstra’s algorithm.

However, the result of these operators is that the search space is reduced, resulting in the risk of the GA converging to bad optima. Therefore, inspiration is taken from the insertion–deletion operator in the work performed by [30], and an operator is introduced to increase the variation in the offspring. The idea behind this variation-inducing operator is that a random coordinate point in the individual is selected, and from the surrounding coordinates a random valid adjacent coordinate is then chosen. Next, the operator selects one of the two variants of this operation with equal probability. In the first variation, the operator replans the path from the chosen adjacent neighbouring point to the end of the path. Moreover, in this operator’s second variation, another random coordinate, after the chosen adjacent coordinate, is selected in the chromosome (individual), and a second valid neighbouring coordinate is then found. The path between these two adjacent coordinate points is then replanned, resulting in new, more diverse routes.

In summary, the modified GA mechanism operation first requires the generation of initial paths using a random walk process, and these initial paths are then repaired using a directed acyclic graph. The evolutionary learning process is then initiated, where two groups of individuals are sampled from the first population. The first group of individuals is selected based on their fitness, and these are the individuals who will be transferred to the new population without any changes. The second group of individuals is selected using a roulette wheel selection process, and these are the parents that will be used to produce the offspring.

The second groups of individuals then undergo a crossover and mutation process to produce new valid solutions, which then replace their parents in forming the second group of individuals. After the reproduction has been completed, the first and second groups of individuals are then merged to form a new generation of individuals, and the breeding process is repeated. The reproduction process is terminated when the algorithm completes a certain number of iterations, and the population is then sorted according to fitness. Finally, the best solution is output by the GA by choosing the solution with the highest fitness.

### 3.3. Multi-Agent System Design

**Figure 2:**
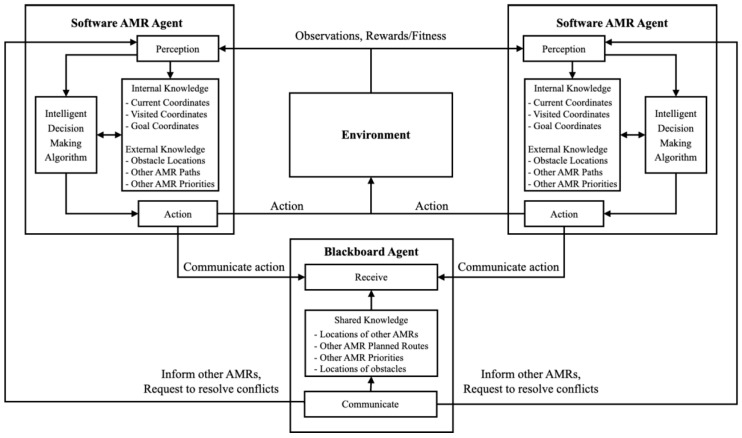
MAS model of the software AMR agents depicting the communication mechanism using the blackboard agent and the interaction mechanisms of the agents with the environment.

For this work, the MAS design is inspired both by the MAS review performed by [40] and similar research, such as the work performed by [41], who designed a MAS for the application of AMRs in a manufacturing environment. Moreover, the MAS literature and theory are also used to define the guidelines and definitions that the MAS design for this work follows. This work builds on top of the work performed on collaborative mobile robots by [41] and uses the surveys on MASs of [40,42] to define two types of software agents that are designed for the MAS, namely the blackboard agent and the software AMR agent. The overall MAS architecture for this work is shown in Figure 2, and the subsequent discussion of the fulfilment of the parameters set out by [43] follows.

Firstly, the software AMR agents are present in the simulation environment and include individual intelligent decision-making algorithms to navigate through the virtual environment autonomously. For this work, the software AMR agents are both reactive, deliberative, and communicate with each other to resolve conflicts (they are “social”). The software AMR agents are reactive as they receive their transport orders from the blackboard agent, triggering the path planning request [41]. Here, the software AMR agents receive inputs from the blackboard agent on changes in the transport orders during execution, such as reordering priorities or changes in workstation locations. Additionally, the AMR software agents are able to observe the environment that they reside in and are able to react to any changes in these observations. Here, the observations within the environment include both the surrounding static obstacles, as well as the dynamic obstacles, such as humans and other AMRs.

The agents also fulfil the deliberative aspect of MASs defined by [43] as they are not limited to only reacting to changes in the environment. Here, the AMR agents use their knowledge about the environment and their own internal intelligent algorithms to plan the subsequent paths to complete the path planning request. Here, the environment provides feedback regarding the suitability of its intelligent decision-making algorithm and allows for the crucial improvement of the agent’s behaviour. Moreover, from Figure 2, it can be seen that each of the AMRs has knowledge about its own transport order and its location in the simulated environment. Here, the internal knowledge, such as the location of transport order, the AMR priority, and the current and historical location information of the current AMR is used as input to the intelligent decision-making algorithm.

Finally, the software AMR agents also exhibit “social” behaviour as they use communication techniques with the blackboard agent to share their internal knowledge. Here, each software AMR agent, therefore, has access to knowledge about external factors, such as the locations of the dynamic and static obstacles, the locations of the other AMRs, and the planned routes and priorities of the other AMRs.

Once the software AMR agents have shared their knowledge, these external factors can also be used to improve the quality of the intelligent decision-making systems that the agents use to define their deliberative behaviour. The “social” behaviour of the agents is a crucial component that allows for communication during the coordination mechanism that is used to avoid conflicts in the multi-robot system [14].

To ensure that this social behaviour is appropriately facilitated in the MAS, the blackboard communication strategy is utilised for this work, as inspired by [41,42]. For this approach, the blackboard agent is created that each agent shares their knowledge with and receives their shared knowledge from. The blackboard agent is not physically present in the simulation and is instead manifested as an abstract entity in the software environment which the software AMR agents receive their instructions from. Unlike the software AMR agents, the blackboard agent is purely reactive and sends requests and information triggered by specific events, such as with an input transport order file being received. The advantage of the blackboard agent and the blackboard communication strategy is that it reduces the communication overheads required in finding a particular agent during the information sharing process.

### 3.4. Multi-Robot Conflict-Resolution Mechanism

**Figure 3:**
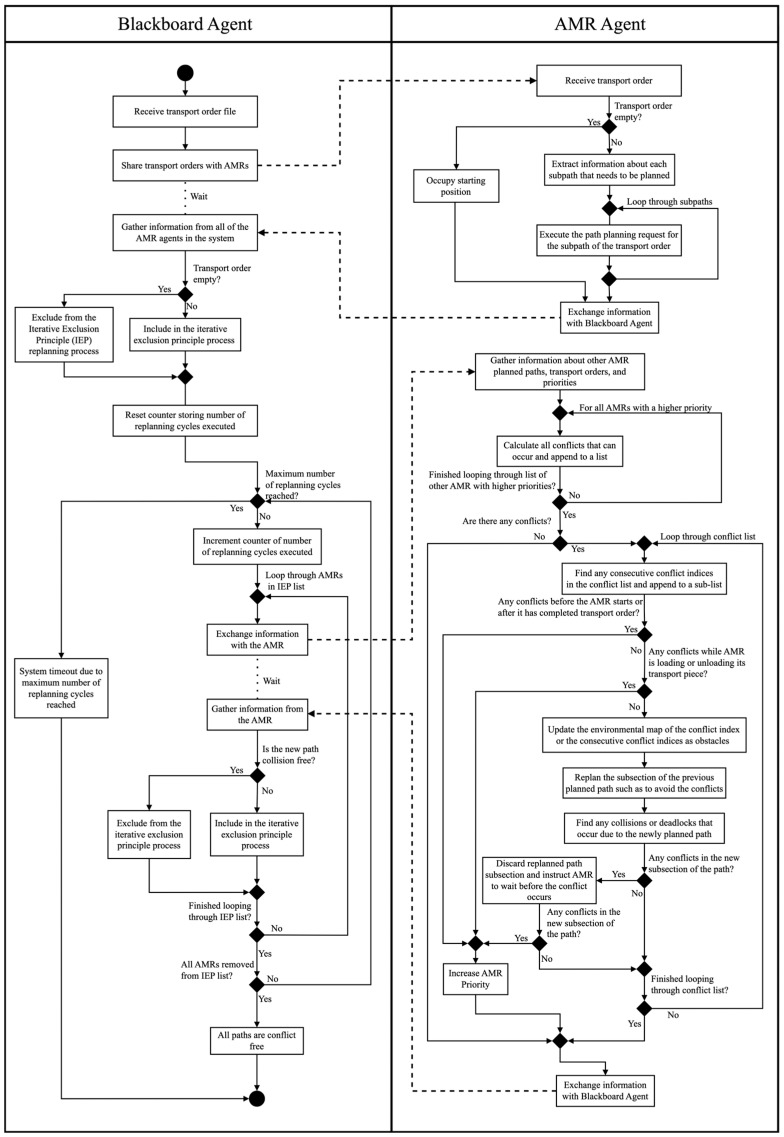
Swimlane diagram summarising the IEP implementation for the MAS agent communication and conflict-resolution mechanism of the dynamic path planning system.

The communication strategy is an essential requirement for resolving the conflicts inherent to multi-robot systems and the interaction between these robots in a confined environment. By using a communication strategy, each of the AMRs is able to acknowledge the priority of the AMRs and appropriately adapt their paths, so as to avoid all of the conflicts in the system while also ensuring that the overall efficiency of the system does not decrease. The multi-robot path planning mechanism of this work is inspired by the IEP proposed by [14], which is an improvement of both the Priority-based Planning (PP) and MPCA algorithms from prior multi-robot conflict avoidance work. The following sub-sections describe the IEP implementation for the MAS agent communication and conflict-resolution mechanism of the dynamic path planning system (refer also to Figure 3).

#### 3.4.1. Receiving and Assigning Transport Orders

For the dynamic path planning system in this work, the algorithm starts when the blackboard agent receives an input containing information about the simulation environment and the software AMR agents. Using this information, the blackboard agent creates the software AMR agents and instantiates the simulation environment using the knowledge about the environmental layout, the number of AMRs, and the home location of these AMRs. Once these parameters have been instantiated, the blackboard agent then waits for transport order inputs. As soon as information about the full, partial, or empty transport orders is received, the blackboard agent then parses the information and creates the objects for the transport orders. The objects for the full, partial, or empty transport orders are then sent to individual software AMR agents for further processing.

#### 3.4.2. Path Generation and Priority Assignment

Each AMR then breaks down the transport order request into requests for the individual paths that must be planned. The individual paths can include the path from the starting location of the AMR to the pickup location, the path from the pickup location to the delivery location, and the path from the delivery location to the home location. The transport orders can also be partial to allow for replanning situations or be empty, where the AMR does not need to plan any paths. Each transport order object sent to the AMRs also includes an associated priority.

For this work, two types of initial path planning requests are experimented with. For the first request type, the AMRs plan their paths independently and rectify all problems during the cyclical conflict avoidance mechanism. For the second, the AMRs plan their initial paths in order of their priority, and lower-priority AMRs contain knowledge about the initial paths planned by the higher-priority AMRs [14]. The approach aims to reduce the number of cycles of the conflict avoidance mechanism, improving the robustness and convergence of the algorithm in heavy-traffic situations.

The software AMR agent then individually plans its paths using its intelligent path planning algorithm, where the knowledge about the higher priority AMRs can be included depending on the path planning logic and algorithm decided on. Each of the AMRs then gathers information about its individually planned paths and transport order priority and uses this as a base for the conflict-resolution system in the following step.

#### 3.4.3. Knowledge Sharing of the Joint Paths

Each AMR then compares its path to the other AMRs’ planned paths for deadlocks and collisions using a Predictive Collision Avoidance (PCA) approach. It is important to note that all software AMR agents with no transport orders assigned to them are excluded from the following process. First, the software AMR agent ranks all of the other AMRs according to their assigned priorities, and then it analyses the joint paths in order to construct a list of coordinates and timesteps at which the deadlocks and collisions with other AMRs occur.

The PCA approach can result in two unique cases that define the rules for if the current AMR is required to replan its path when taking the priority system into account. The first case arises when the other software AMR agent has a lower priority than the current software AMR agent. The second case arises if the other software AMR agent has a lower priority than the current software AMR agent; however, the collision occurs at one of the significant locations of the lower-priority software AMR agent. If neither of these cases are present, then the current software AMR agent is excluded for the IEP process.

#### 3.4.4. Inclusion and Replanning Process

For this work, the replanning is completed using a cyclical collaborative conflict avoidance planning mechanism. At the start of each cycle, each software AMR agent receives the most recent planned paths of the software AMR agents that are included in the IEP. Using this knowledge, the software AMR agent then uses the PCA approach to construct an updated list of conflicts that it is responsible for. The software AMR agent then iterates through this list of conflicts and breaks the conflicts into sub-groups consisting of either individual conflicts or consecutive conflicts that it can solve in one replanning attempt.

Each of the conflict locations is marked as a time-dependent obstacle, and the software AMR agent is not allowed to occupy this area during the replanning process. Once the replanning is completed, the software AMR agent compares the newly planned path segment with the other AMR paths in the specific time window using the PCA approach. If the newly planned path segment results in another conflict, then the path segment is discarded, and the AMR is instructed to wait to avoid the conflict. If the waiting results in another conflict, the AMR priority is updated, and the conflict-resolution responsibility is transferred to another software AMR agent in the subsequent cycle of the IEP.

The blackboard agent waits for each of the software AMR agents to complete their conflict avoidance tasks and receives information from the software AMR agent about either the success or failure of this request. If the software AMR agent succeeds in resolving all of the conflicts that it is responsible for, then the blackboard agent excludes it from the following cycle of the IEP algorithm. However, if the software AMR agent is the subject of a conflict avoidance transfer or if it has failed to resolve a conflict, then it is included in the following cycle of the IEP. This iterative process is then repeated until all mobile robots have been excluded from the IEP algorithm, and no conflicts occur in the system, or the algorithm times out due to extreme congestion [14].

## 4. Evaluation and Results

Three different evaluation experiments were conducted to evaluate the performance of the developed dynamic path planning system for autonomous mobile robots using a multi-agent system approach. The first experiment evaluated the performance of the modified GA path planning algorithm against an implementation of Dĳkstra’s algorithm, as well as a reinforcement learning (RL)-based path planning algorithm. The second experiment evaluated the scaling ability of the developed path planning system under an increasing number of autonomous mobile robots, and the third experiment evaluated the performance of the system under different disturbance scenarios. For this research, the focus was on small-to-medium-sized manufacturing environments with medium-level computer hardware affordable enough to be installed as an on-premise infrastructure in most SMEs. Here, the idea was that dynamic path planning should not be trumped by the costs of purchasing and operating a costly dedicated computer. For this approach, it was envisaged that edge devices, including the onboard AMR computing hardware and localisation sensors, send the data for path planning requests directly to a decentralised device on the FMS premises that are dedicated to computing the conflict-free paths for the AMRs. Therefore, all experiments were conducted on a machine with a 3.8 GHz i7 processor with 16 GB of RAM and a GTX1050Ti GPU utilising 4 GB of VRAM. For the AMR, the Neobotix MP700 AMR platform was chosen due to its versatility in use cases and the availability of the model file for the Matlab R2022a RobotScenario simulation environment. For this work, the cell size for the CBD was selected as 1 m × 1 m according to the size of the MP700 platform and to allow for slight deviations when the AMR executes turns.

The following sub-sections briefly explain the experiments and discuss the results of each.

### 4.1. Evaluation Against Other Path Planning Algorithms

**Figure 4:**
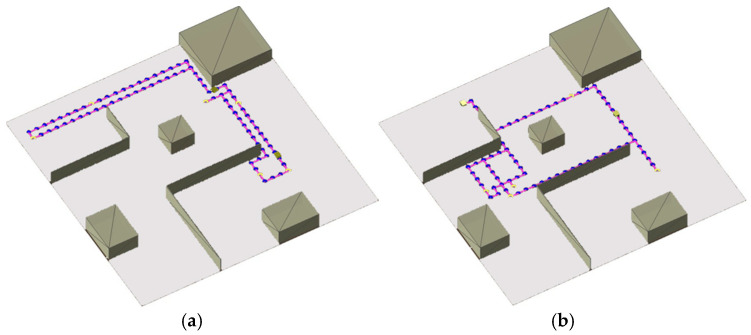
Visualisation of the first 30 m × 30 m environment used for evaluation. (**a**) An example of a combination of tasks, and (**b**) another example of a combination of tasks. The blue dots and purple lines denote the planned coordinate trajectory of the AMR, and the yellow dots denote the coordinates of interest for this trial.

The first evaluation was conducted in a 30 m × 30 m simulated environment using only two AMRs. This environment represents an indoor scene with walls and scattered obstacles. A visualisation of the environment used for this experiment is shown in Figure 4, depicting two configurations of different points of interest (two random test trials). The dotted lines denote the planned coordinate trajectory of the AMR, and the yellow dots denote the coordinates of interest for this trial. For this experiment, twelve coordinates of interest were generated prior to the experiment, and during each trial, six random coordinates were sampled and assigned to either AMRs to generate a set of unique transport orders. The first three coordinates were assigned to the first mobile robot, where the first coordinate denotes the starting point of the first mobile robot, the second coordinate denotes the pickup location coordinate, and the third coordinate denotes the drop-off location coordinate. Finally, the transport order was created by combining these three coordinates such that the first mobile robot is instructed to return to its starting locations after visiting the pickup and drop-off locations. The last three coordinates that were sampled from the set were then similarly assigned to the second mobile robot. For this experiment, 100 trials were conducted.

The developed GA-based path planning algorithm was compared against two other classes of algorithms: (1) Dijkstra’s algorithm, which is a well-established graph-theory approach, and (2) a Reinforcement Learning (RL)-based approach. Dĳkstra’s Algorithm was implemented to use as a baseline for comparing. RL is a recent AI-based approach that has received a lot of attention in its application to the global path planning problem. Recently, RL has been combined with deep learning to allow for processing complex inputs using value function approximation. A promising architecture for improving the learning of RLs includes the Double Deep Q-Network algorithm (DDQN), a reinforcement learning algorithm that builds upon the Deep Q-Network (DQN) by addressing the issue of Q-value overestimation. In essence, DDQN uses one network to select the best action and another to evaluate its value, rather than relying on the same network for both [44]. Other improvements to the learning capability of RLs as derived from the literature include Convolutional Neural Networks (CNNs) [45] and Gated Recurrent Units (GRUs) [46,47]. GRUs are a type of neural network that efficiently captures patterns in sequential data by using gates to control the flow of information and maintain memory over time and therefore provides a powerful approach for handling spatio-temporal data. For this research, an RL algorithm based on a DDQN GRU–CNN architecture was therefore used to compare with the developed GA approach for path planning.

This work aimed to design a dynamic path planning system that exhibits good performance in terms of the path quality generated by intelligent path planning algorithms while maintaining reasonable execution times. Therefore, for this work, several individual criteria have been identified to provide quantifiable measures for comparison. The selected evaluation criteria and their reasons for inclusion are presented in Table 1, together with descriptions of the weighted scoring scheme used for each criterion. A weight was assigned to each criterion denoting its importance, where criteria that are more favourable than others could be prioritised. Therefore, for the final algorithm comparison, a single score could be calculated for each algorithm, and the algorithm with the highest score over all the criteria was identified.

The results from the algorithm comparison experiment are presented in Table 2. A failure occurs if the algorithm is unable to plan any sub-path during the path planning task. Both Dijkstra’s algorithm and hybrid GA performed optimally in this regard with zero failures in the 100 trials, while the RL algorithm failed to plan a path for 9 out of the 100 trails. The number of collisions should be minimised to avoid system failure. The hybrid GA was the only one to completely avoid collisions, outperforming both Dijkstra and RL. A deadlock is a situation where two AMRs cannot continue their pre-planned paths without colliding. Again, the hybrid GA was superior, eliminating all deadlocks, while RL performed better than Dijkstra but still encountered deadlocks. For the weighted path length performance criteria, Dijkstra’s algorithm served as the baseline with a score of 1 (indicating the weighted average path length for AMRs divided by Dijkstra’s path length). A shorter path length indicates a more efficient path. The RL with DDQN GRU–CNN Architecture had a weighted path length score of 1.09, meaning its paths were slightly longer than Dijkstra’s. The hybrid GA scored 1.01, indicating its paths were very close to Dijkstra’s optimal length, making them more efficient than the RL algorithm’s paths. Path smoothness for mobile robot paths is an important criterion for evaluating the quality of generated paths and determines the energy usage of the AMR, which should be minimised. The smoothness of a generated path was measured by calculating the deflection angle between any three waypoints in the trajectory. Dijkstra’s algorithm served as the baseline with a path smoothness score of 1. The RL with DDQN GRU–CNN Architecture had a score of 2.64, suggesting its paths were significantly less smooth than Dijkstra’s, implying higher energy usage. The hybrid GA achieved a score of 0.95, which means its paths were smoother than Dijkstra’s. Path overlap score needs to be minimised to reduce the risk of replanning, which increases execution time. For the execution time, Dijkstra’s algorithm had an execution time score of only a few milli-seconds, which rounded to 0. The hybrid GA showed faster execution times than the RL algorithm, which is important for reactive and fast planning and replanning (although slower than for Dikstra’s algorithm, it is still acceptable from a practical perspective).

The results clearly indicate that the hybrid GA significantly outperforms both Dijkstra’s algorithm and the RL with DDQN GRU–CNN Architecture across almost all identified evaluation criteria. The hybrid GA excelled in critical areas such as eliminating failures, collisions, and deadlocks, which are crucial for the robustness and reliability of autonomous mobile robots in a manufacturing environment. Its path length was very close to optimal (Dijkstra’s), and it produced significantly smoother paths with less overlap, contributing to efficiency and reduced replanning. It also demonstrated faster execution times compared to the RL approach. Dijkstra’s algorithm serves as a baseline and shows optimal path length performance but struggles with multi-agent scenarios, exhibiting collisions and deadlocks. The RL with DDQN GRU–CNN Architecture, despite being a recent AI-based approach, performed the worst overall, showing a high number of failures, collisions, and deadlocks, as well as longer and less smooth paths with more overlap compared to both Dijkstra and the hybrid GA. While RL combined with deep learning like DDQN, CNNs, and GRUs is promising for handling complex and spatio-temporal data, its implementation in this context did not yield superior results for dynamic path planning for autonomous mobile robots compared to the developed GA approach.

In summary, the evaluation demonstrates the superiority of the hybrid GA for the developed dynamic path planning system, achieving excellent path quality and maintaining reasonable execution times, particularly in terms of safety and efficiency, by avoiding collisions and deadlocks, and generating efficient and smooth paths. This aligns with the research’s aim to design a system with good performance while maintaining reasonable execution times [44,48].

### 4.2. Evaluation Under Increasing Number of AMRs

**Figure 5:**
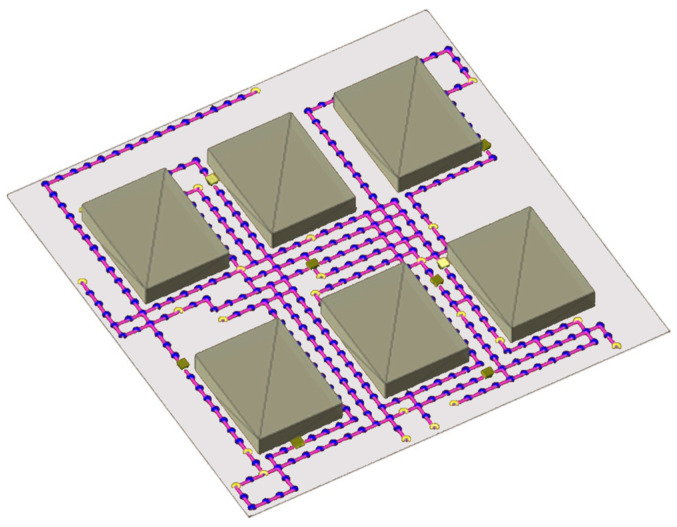
Visualisation of the 30 m × 30 m environment with 8 AMRs concurrently traversing their paths using the multi-AMR collaborative path planning mechanism. The blue dots and purple lines denote the planned coordinate trajectory of the AMR, and the yellow dots denote the coordinates of interest for this trial.

The second evaluation experiment was conducted to evaluate how the chosen intelligent GA path planning algorithm scales under an increasing number of AMRs. The environment shown in Figure 5 was used as it resembles the confined workspace layout standard in matrix production facilities.

For each experiment used for the evaluation, fifty coordinates of interest were generated before testing, and three random coordinates were sampled from this set for each AMR during testing. The first coordinate denotes the starting point of the AMR, the second coordinate denotes the pickup location, and the third coordinate denotes the drop-off location. The transport order for each AMR was created by combining these three coordinates such that the mobile robot is instructed to return to its starting locations after visiting the pickup and drop-off locations. Furthermore, this process of assigning transport orders and planning paths was repeated 100 times for each number of AMRs until a failure is recorded. Figure 5 illustrates the transport order assignment for the case of eight AMRs concurrently moving in the environment.

**Figure 6:**
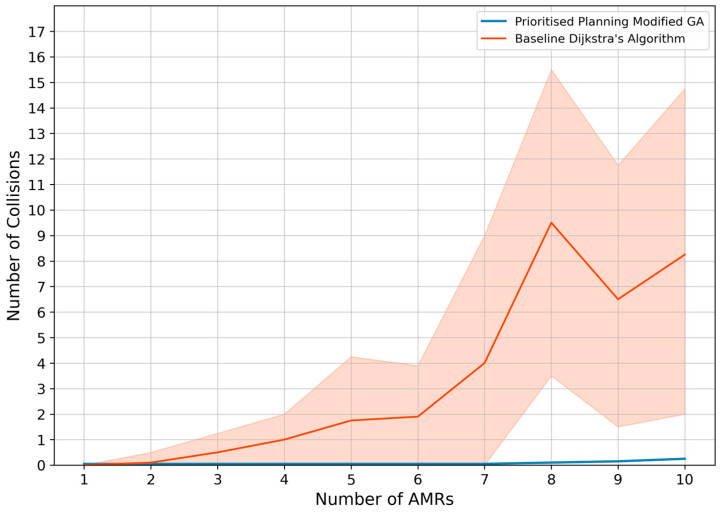
The number of collisions as a function of the number of AMRs to evaluate the dynamic path planning scalability, with the baseline shown in orange and the proposed dynamic path planning system shown in blue (mean values). The orange area indicates the standard deviation around the mean values for the baseline Dijkstra’s algorithm.

**Figure 7:**
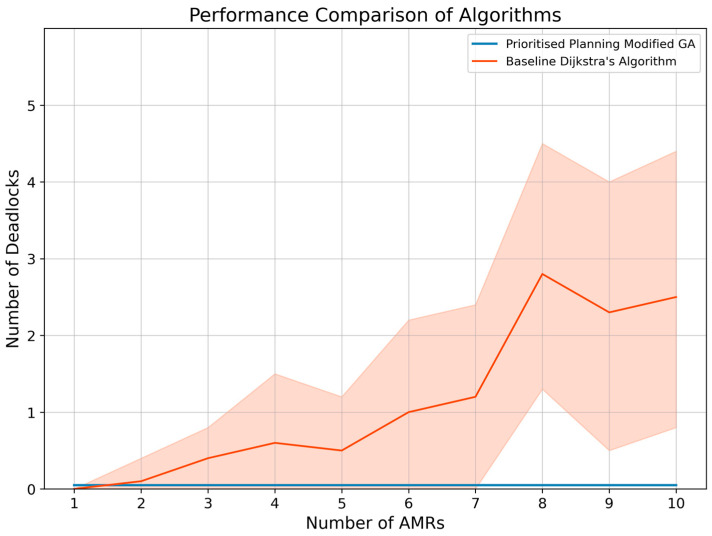
The number of deadlocks as a function of the number of AMRs to evaluate the dynamic path planning scalability, with the mean values for the baseline shown in orange and the mean values for the proposed dynamic path planning system shown in blue (on the horizontal axis since the number of deadlocks for GA-based path planning system remained zero with increasing number of AMRs). The orange area indicates the standard deviation around the mean values for the baseline Dijkstra’s algorithm.

The complexity of the problem and finding optimal paths for each AMR increases exponentially with the increase in the AMRs. The increase in complexity is specifically due to an increase in the number of dynamic obstacles each AMR has to face in finding each of its three paths. Figure 6 illustrates the number of collisions as the number of AMRs increases, comparing the proposed dynamic path planning system (blue) with the baseline Dijkstra’s algorithm (orange). As the number of AMRs grows, the baseline approach shows a steep increase in collisions due to the higher complexity of navigating around an increasing number of dynamic obstacles. In contrast, the proposed GA-based system maintains a near-zero collision rate, with only a single collision occurring at 10 AMRs in one of the 1000 trials, demonstrating superior collision avoidance performance and scalability. Figure 7 shows the number of deadlocks as a function of the number of AMRs. The baseline Dijkstra’s algorithm exhibits a gradual rise in deadlocks as the number of AMRs increases, highlighting its limited ability to handle congestion in dense environments. The proposed GA-based dynamic path planning system completely eliminates deadlocks across all trials, regardless of the number of AMRs, with its performance shown as a flat line on the horizontal axis. This result confirms the effectiveness of the proposed approach in preventing deadlocks even under increasing system complexity.

Here, the mean number of collisions and deadlocks for the 100 trials per number of AMRs (up to 10 AMRs) are recorded for the chosen modified GA and compared to a baseline Dijkstra’s algorithm. The findings show that the baseline Dijkstra algorithm has an increasing number of collisions and deadlocks when more AMRs are introduced into the system. The dynamic path planning system implementation can successfully avoid all deadlocks in the system; however, the system was unable to avoid a single collision for 10 AMRs during one of the 1000 trials.

However, failures in the system are expected for a substantial number of AMRs, because an extreme amount of congestion occurs in the environment, and a particular configuration of transport orders will result in the system failing. Therefore, compared to Dijkstra’s algorithm for path planning, the proposed path planning system scales exceptionally well, with a guarantee of no deadlocks or collisions for up to nine AMRs in an environment with confined space in the moveable area. The reason for the collision is explained as follows. The IEP collision and conflict avoidance algorithm performs a cyclical procedure where it cycles through each of the AMRs and checks their priorities before deciding to adapt their paths. The adapted paths are then propagated to the other software agents, and the cyclical process is repeated until all paths are conflict-free. Depending on the conflict scenarios, the cyclical process can be repeated many times, resulting in longer run times. Therefore, a design choice was to limit the number of these cyclical processes to 50, where the system then stops and the AMRs follow their paths. To pragmatically resolve the conflict in a timely manner, the AMR that would encounter the conflict should wait upon detecting another AMR, and once the congestion has cleared, the AMRs should collaboratively replan their paths.

More specifically, a practical implementation of this is that as the number of AMRs increases to a large amount and congestion in the environment reaches an extreme, the dynamic path planning system might not be able to produce conflict-free paths immediately, and the initially planned paths might have to be regenerated several times. As a result, the algorithm’s run time becomes unstable, and it is not guaranteed that optimal joint paths can be calculated quickly for a substantial number of AMRs. However, this phenomenon regarding the run time is only applicable to the extreme case, and the overall dynamic path planning system robustness performance yields favourable results. The algorithm scalability results further showed that the algorithm could plan conflict-free paths for up to nine AMRs in a highly congested environment.

Moreover, the algorithm run times also produced favourable results where the system scaled linearly with an increase in AMRs (from ±2 s average for 2 AMRS to ±17.5 s for 10 AMRs). Once the system reaches an extreme amount of congestion, the run times become much longer. However, such a system with minimal floor space and a considerable number of AMRs does not represent a real-world scenario and merely represents a stress test scenario of the dynamic path planning system.

In summary, although the proposed GA-based multi-agent path planning system exhibits favourable scalability and robust conflict avoidance performance for small-to-medium-sized AMR fleets, several limitations emerge when the system is subjected to higher robot densities. As the number of AMRs increases, congestion effects escalate nonlinearly, leading to a significantly higher frequency of dynamic conflicts. Under these conditions, the iterative exclusion mechanism must perform repeated replanning cycles, which substantially increases computational demands and, when iteration limits are reached, may result in residual collisions or suboptimal path solutions. Furthermore, while the average computational run time scales approximately linearly up to nine AMRs, highly congested scenarios produce unstable run times as the negotiation process between agents becomes prolonged. This effect is particularly pronounced in environments with continuously changing transport orders, where frequent replanning may delay path generation and negatively impact system throughput. In addition, the coordination overhead associated with a fully distributed, communication-intensive architecture becomes more significant as fleet sizes grow. Without additional hierarchical coordination or clustering strategies, the exchange of path information and priority negotiation among a very large number of agents may impair scalability.

These findings indicate that the current approach is well suited for small to moderately sized fleets operating in constrained environments but that further algorithmic refinements—such as hierarchical planning, hybridised optimisation methods, or advanced cooperative control schemes—will be necessary to maintain robustness and real-time responsiveness in substantially larger or more congested systems.

### 4.3. Evaluation Under Various Disturbance Scenarios

The third experiment evaluated the performance of the developed path planning system under various disturbance scenarios by using a realistic simulation model of an Industry 4.0 production environment. For this work, the system is validated in a simulation model of a logistics learning factory. Learning factories provide safe and industry-oriented production environments where these new technical solutions can be evaluated and verified before being seamlessly transferred into industrial practice. The logistics learning factory environment used is fully equipped for producing multi-variant city scooter models with state-of-the-art planning and control methods. Moreover, all workstations and logistics infrastructure are entirely mobile, allowing for the rapid reconfiguration of the factory layout to accommodate the proposed production processes.

**Figure 8:**
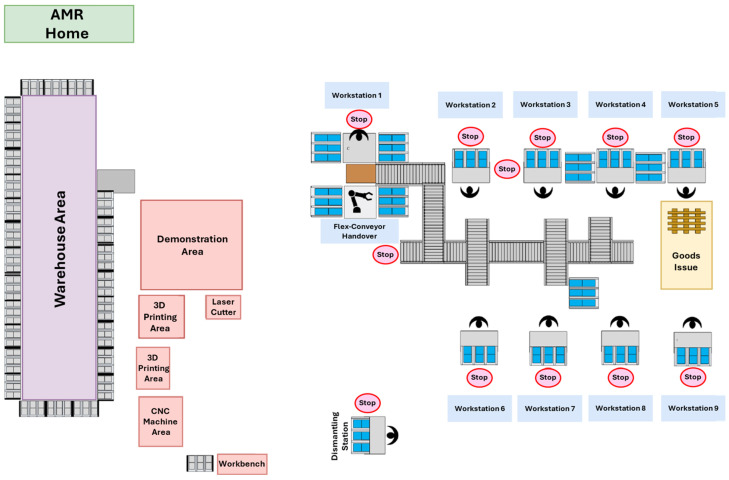
Current layout of the learning factory production facility, depicting the workstations, the chosen AMR home for this thesis, and the demonstration areas that are off limits.

**Figure 9:**
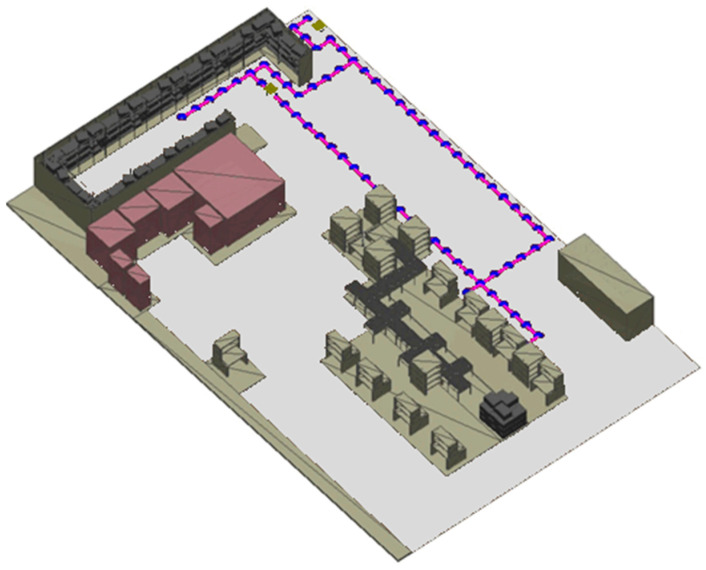
Visualisation of the 3D simulation environment of the learning factory developed in Matlab. The blue dots and purple lines denote the planned coordinate trajectory of the AMR, and the yellow dots denote the coordinates of interest for this trial.

The workstations at the learning factory are also equipped with tablet PCs and several IoT devices to allow real-time data monitoring and transfer between the intelligent objects in the learning factory. Therefore, the learning factory is selected for the single-case study in this research due to several reasons: (i) it is a realistic representation of an Industry 4.0 production system, (ii) it allows for the safe development of new technologies and prototypes, and (iii) the appropriate enabling technologies are available to support the development of intelligent systems. The current layout of the learning factory facility used for the validation is depicted in Figure 8. A three-dimensional simulation environment of the learning factory has been developed in Matlab R2022a to provide a simulated environment for validating the dynamic path planning system (refer to Figure 9).

The work performed by [49,50] was used to define several flexibility types associated with autonomously controlled intralogistics. The most important flexibility types associated with the practical implementation of the dynamic path planning system are material handling flexibility and control program flexibility. Here, material handling flexibility is defined as the number of allowed paths between the different workstations and the material handling system’s capacity to transport material between the different workstations effectively. The flexibility parameter for this type of flexibility is thus the number of transport paths. Moreover, control program flexibility is the ability of the dynamic path planning system to operate nearly uninterrupted during the production phase.

Additionally, ref. [49] also documents the disturbances common in Industry 4.0 environments that are relevant to the corresponding flexibility parameters. To validate the dynamic path planning system developed in this study, the following validation case scenarios have been defined that are aligned with these disturbances:

(1) Scenario 1: Missing part at workstation;
(2) Scenario 2: Excess supply at workstation;
(3) Scenario 3: Addition of a static obstacle;
(4) Scenario 4: Failure of transport system;
(5) Scenario 5: Addition of a dynamic obstacle;
(6) Scenario 6: Machine breakdown at workstation.

In this research, the run time of the dynamic path planning system was evaluated before and after each of the above disturbances are introduced. The run times are required to be consistent and should not take too long, as one of the practical requirements of the system is that it should be responsive and should not interrupt the execution of the FMS.

For each disturbance scenario, the path quality of the dynamic path planning system was evaluated for each of the AMRs under the different scenarios. The path quality metrics include the paths’ lengths, the paths’ smoothness, and the total number of overlaps between the AMR paths. The path planning system should be able to consistently optimise its output based on these three metrics and produce paths that are not detrimental to the overall efficiency of the FMS. Finally, an important unit of analysis was investigating the interplay between the robustness and flexibility of the dynamic path planning system to conclude its practicality.

*Scenario 1: Missing part at workstation*

For the first scenario, the short-term disturbance of a missing part at a workstation is simulated. The first AMR starts at its home position and receives a transport order to deliver a packaging board to workstation 5. However, while the first AMR executes its transport order, the worker at workstation 2 realises that a smart bin of screws has been depleted. A rushed order is then placed to prioritise the delivery of the batch of ISO screws, as this might delay the overall production time. After the rush order has been triggered, the AMRs collaboratively plan their paths to ensure that the second AMR receives priority. After both AMRs have completed their transport orders, they are then required to return to their home positions. The time the second transport order is triggered is sampled from a uniform random distribution within the range of 1 and 80 s to ensure that the majority of the transport orders include both AMRs.

The results for 50 simulation runs are summarised in Table 3 (where the smoothness score was calculated with Equation (2)). Here, the run times remain stable throughout the progression of the simulation, with the mean execution times reported as 1.4941 s and 2.3272 s, respectively. The execution time before the disturbance only includes the path planning time for the first AMR, and this has a standard deviation of only 0.1249 s. After the disturbance, the execution time increases as this includes the path planning of both AMRs. Here, the standard deviation of 0.5480 s is expected as the IEP has to be applied to the robots avoiding collisions at different locations. Finally, the path planning metrics also show stable behaviour, where there is a larger variance in the metrics for the first AMR as it has to give the second AMR priority under different circumstances.

*Scenario 2: Excess supply at workstation*

For the second simulation scenario, the short-term disturbance of excess material at a workstation is simulated to investigate how the dynamic path planning system would react to non-conventional transport orders. The first AMR receives a transport order at the start of the simulation to transport a batch of four rear forks for the Flex Blue scooter model to the commissioner workstation. Similarly to the previous experiment, the disturbance time is sampled from a uniform random distribution with a range between 1 and 60 s. However, the worker at workstation 3 realises that, by mistake, two batches of hexagonal nuts had previously been delivered. The task is assigned to the second AMR as the first AMR is still completing its transport order, and the two AMRs collaboratively have to replan their paths. Similarly to the previous scenario, the execution time before the disturbance (1.3013 ± 0.2027 s) is much shorter than the execution time after the disturbance (2.1651 ± 0.4612 s), as initially, only the first AMR plans its path, and after the disturbance, collaborative planning occurs. Moreover, as the planned paths for this scenario are on average shorter than for the previous scenario, as shown in Table 4, the mean execution are also less. However, this scenario also yielded very stable execution times before and after the addition of the disturbance, with the larger standard deviation being attributed to the difference in length of the first AMRs planned path due to the completion of its transport order.

*Scenario 3: Addition of a static obstacle*

The third scenario investigated how the proposed dynamic AMR routing system would react to a static obstacle added to the environment during run-time. Both AMRs receive their transport orders simultaneously and collaboratively plan their paths. However, during the traversal of the collaboratively planned paths, one of the AMRs detected an obstacle that was not present during the initial path planning. Both AMRs are then required to replan their paths such that the AMR that is obstructed by the obstacle can reach its target. For this experiment, the location of the static obstacle was the control variable, and the location of the static obstacle was randomly generated during each simulation run such that a collision is guaranteed to occur.

For this scenario, a good representation of algorithm stability is if the actual simulation result is very similar to the planned paths before the disturbance occurred. Comparing the results in Table 5, the result due to the disturbance and the planned paths before the disturbance are very similar. Here, some statistical outliers are present in the distance travelled and the smoothness score, where certain collisions with the static obstacle occurred in such a way that it resulted in a possible conflict between the AMRs. Therefore, the AMRs had to plan paths that were longer to collaboratively avoid conflicts with each other and as a result, the smoothness score of the AMRs were also increased.

*Scenario 4: Failure of transport system*

For this scenario, the static obstacle introduced to the environment was one of the AMRs that has broken down during run-time. To further explain this scenario, at the start of the simulation, both AMRs were assigned transport orders, and after a certain amount of time, the first AMR breaks down, resulting in an unexpected obstacle on the production floor. The time the first AMR breaks down is sampled from a uniform random distribution and can be between 10 and 60 s. Furthermore, the transport orders for the first AMR were to deliver five metal footboards to workstation 2, and the second AMR was required to deliver a batch of ten counter screws to workstation 3.

As with the previous scenario, the dynamic path planning system displays robust behaviour to the second type of static obstacle. The run-times align with what was observed previously, with an acceptable mean and standard deviation for the reactive behaviour of the second AMR (execution time before the disturbance was 2.1184 ± 0.0956 s, and 0.8291 ± 0.2351 s after the disturbance). Furthermore, the path metrics displayed in Table 6 also display favourable behaviour for the second AMR. The second AMR does not display a significant variation in the distance or smoothness of the paths it plans after detecting the faulty AMR, which concludes that the system works successfully. Moreover, the path metric results for the first AMR can mainly be ignored as the mean and standard deviation of the travel distance occur as a result of the time of breakdown of the AMR.

*Scenario 5: Addition of a dynamic obstacle*

As the dynamic path planning system has shown promising results in successfully adjusting its planned paths in light of both a static obstacle and an AMR breaking down, the next scenario investigated how the dynamic path planning system would react to adding a dynamic obstacle to the environment. Here, a dynamic obstacle in the form of a human worker was added to the environment. The human worker starts in the middle of the warehouse area and carries a workpiece directly to the conveyor’s hand-off point opposite the Commissioner Workstation. The worker’s walking path is first planned from the starting point to its ending point using a shortest path approach, and then stochasticity is introduced to model an accurate representation of a person deviating from their initial path. Here, two random points are chosen in the human’s path, and the worker deviates from the original path between these two points.

For this experiment, the simulation was also repeated 50 times; however, as the worker follows a different path during each simulation run, it was also necessary to vary the time the human starts walking to ensure a collision would occur. This parameter was sampled from a uniform random distribution with a minimum and maximum time of 0 and 10 s. Before the start of each simulation trial, a number of random time and random path combinations were generated until a combination was found that would result in a collision with an AMR. Furthermore, to increase the complexity of the scenario, the AMRs have to plan around the dynamic obstacle once a possible collision has been detected, where the worker in the simulation is instructed to stand still until the AMR has passed around it.

From Table 7, it can be seen that the dynamic path planning system effectively replans the AMR paths, even for scenarios with multiple collisions, and can robustly react to the addition of a dynamic obstacle to the environment. Furthermore, for this scenario, the AMR path metrics remain relatively stable, with the standard deviation in the path distance and path smoothness being attributed to the AMRs having to replan around the dynamic obstacle.

*Scenario 6: Machine breakdown at workstation*

**Figure 10:**
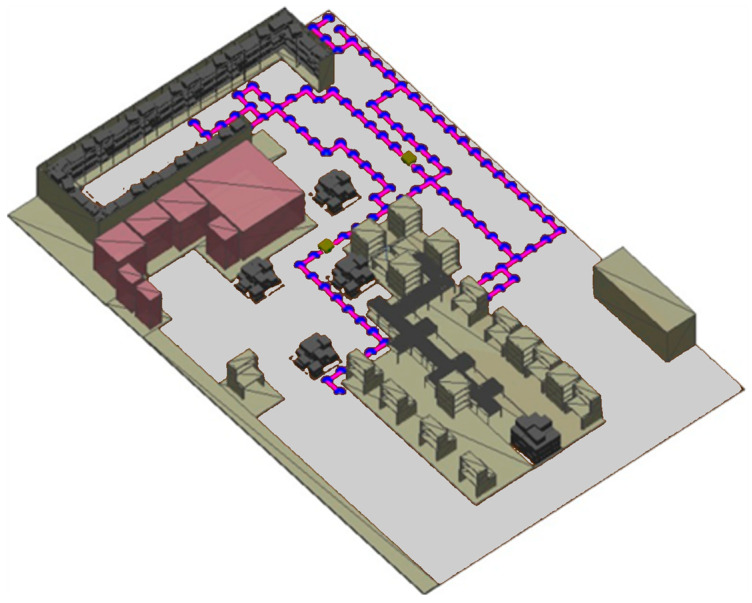
Visualisation of the additional static obstacles in the form of boxes introduced into the learning factory simulation environment for disturbance Scenario 6.

In this scenario, the final validation criteria of reactively adapting previously planned paths to a workstation becoming unavailable during run-time are tested. For this simulation scenario, the first AMR is required to deliver a batch of splash guards to the commissioner workstation. Furthermore, the second AMR is required to deliver a batch of wheels to workstation 2. Additionally, to illustrate that the dynamic path planning system can plan complex paths in this environment, some obstacles in the form of boxes are scattered around the environment, as shown in Figure 10. After the failure at workstation 2 has been detected, the dynamic scheduling system reassigns the transport order to be delivered to Flex workstation 1. Consequently, the AMR updates its planned path during run-time to adjust for this change. Here, the time the worker at workstation 2 signals that the workstation is no longer available was sampled from a uniform random distribution with a range between 1 and 60 s.

The results for the final scenario are shown in Table 8. As with all of the previous scenario results, the dynamic path planning system displayed good reactivity in terms of run times before (1.9909 ± 0.0841 s) and after (2.6996 ± 0.6962 s) the signal was given that the workstation became unavailable. Additionally, the added complexity in the environment in the form of scattered boxes do not cause the dynamic path planning system’s planned paths to deteriorate, further showing favourable performance. Furthermore, the smoothness score for the first mobile robot is nearly twice that of the second AMR. However, this is expected as the free-moving space for the first AMR is obstructed by boxes, and it needs to traverse around these obstacles when it moves to the new workstation.

*Summary*

The experimental evaluation across six representative disturbance scenarios confirmed that the proposed dynamic path planning system consistently enabled the autonomous mobile robots (AMRs) to generate collision-free and efficient paths under a variety of operational conditions. In all scenarios, the pre-disturbance planning times ranged from 1.3 s to 2.1 s, while post-disturbance times remained below 2.7 s, confirming that the system operates within practical real-time limits. The system consistently adjusted to machine breakdowns, obstacle introduction (static and dynamic), re-prioritised tasks, and human interaction without loss of stability. Across scenarios, travel distances remained between 65 and 100 m, smoothness scores between 24 and 45 rad, and path overlaps below 25 m, ensuring safe, efficient, and non-congested routing. No irregularities, skipped processes, or erratic behaviours were observed during animation and trace validation, confirming the reliability of the approach. Collectively, these results demonstrate that the developed dynamic path planning system maintains robust performance with respect to execution time, path efficiency, and system resilience under a broad spectrum of disturbances. The findings substantiate the practical feasibility of deploying this technology in Industry 4.0-oriented flexible manufacturing systems, where adaptability to uncertainty and responsiveness to disruptions are critical determinants of operational performance.

## 5. Conclusions

Developing dynamic path planning systems will assist organisations in fully unlocking the capabilities of AMRs in the flexible environments of Industry 4.0 production facilities. Moreover, by presenting a decentralised and fully autonomous design, this work enables Logistics 4.0 by making the AMRs intelligent objects guided by their software agents in the digital model of the environment. Consequently, the intelligent control of AMRs allows for a more widespread and versatile use of the technology, reducing labour costs associated with intralogistics and ensuring that intralogistics activities can be completed with higher consistency.

The main contribution of this study is a hybrid GA formulation that is deployed in a multi-agent approach for dynamic path planning of AMRs in dynamic production environments. This allows AMRs to reactively adapt to the disturbances in a flexible manufacturing environment and can generalise to different factory layouts. This research builds on, extends, and integrates various previous research studies related to the multi-AMR path planning problem. The dynamic path planning system presented in this paper advances the state of the art by combining a novel hybrid Genetic Algorithm with a decentralised Multi-Agent System based on the Iterative Exclusion Principle. In contrast to conventional hybrid methods that often initialise GA populations randomly or with simplistic heuristics, our approach integrates Dijkstra-informed initialisation and repair, producing feasible, high-quality starting paths that accelerate convergence. Unlike most GA-based multi-robot methods, our fitness function explicitly considers path overlap as a safety metric, rather than focusing solely on path length or clearance, which improves collision avoidance and reduces conflict-resolution overhead. Additionally, the introduction of domain-specific mutation operators distinguishes this work from prior GA approaches that suffer from infeasible solutions due to generic operators. Compared with decentralised MAS approaches such as those of [14], which rely on simple graph search for path planning, our method integrates advanced GA-based path planning with the IEP mechanism, resulting in superior deadlock and collision avoidance without the computational burden of centralised schemes like Conflict-Based Search (CBS) [16,25]. Furthermore, unlike reinforcement learning methods that require time-consuming retraining and struggle with layout changes, the proposed GA-based system generalises immediately to new environments and disturbances without additional training. The combined approach was validated in a realistic flexible manufacturing setting, where it consistently planned conflict-free paths for up to nine AMRs, handling static and dynamic obstacles and responding effectively to disruptions. These findings demonstrate that the proposed framework provides a scalable, adaptive, and practically deployable solution in dynamic, confined industrial environments with small-to-medium-sized mobile robot fleets. More research is required to further improve for application in substantially larger or more congested systems.

This work was only confined to the implementation in a simulated environment, and as a result, the system could not be evaluated at the hardware level in a physical environment. Since the work did not explore decentralisation at the hardware level, the communication protocol over the shared network was not selected. More research is required in this aspect, where the appropriate network protocol and agent communication language and ontology must be integrated into the FMS environment. Future research could also focus on practically testing the AMRs in the physical environment, mounted with manipulator arms to test the effect of variable loading and unloading times for the dynamic path planning of AMRs. Another limitation is that this work only focused on comparing GA and Dĳkstra’s algorithms. Future research could explore other hybrid approaches such as Ant Colony Optimisation, Particle Swarm Optimisation, simulated annealing, tabu search, as well as reinforcement learning.

## Figures and Tables

**Table 1:** Evaluation criteria for the comparative evaluation.

Factor	Weight	Reason for Inclusion	Scoring Scheme
Weighted Path Length	3	A shorter path length indicates a more efficient path.	The score is the weighted average path length of the two AMRs, with a 60% weighting assigned to the prioritised AMR and a 40% weighting assigned to the other AMR. A single normalised score is then obtained by dividing the weighted average path length for the AMRs by the path length obtained by Dĳkstra’s algorithm.
Weighted Path Smoothness	3	The path smoothness determines the energy usage, which should be minimised.	The score is the weighted average path smoothness, with a 60% weighting assigned to the prioritised AMR and a 40% weighting assigned to the other AMR. The final path smoothness score is obtained by dividing the weighted average path smoothness of the AMRs by the path smoothness obtained by Dĳkstra’s algorithm.
Total Path Overlap	3	The path overlap score is represented by the total overlap between AMR paths and needs to be minimised to reduce the risk of replanning, which also increases the execution time.	The score is the total path overlap score obtained by dividing the total overlap between the AMR paths by the total path length. The final path overlap score value is obtained by dividing the path overlap score by the total path overlap obtained by Dĳkstra’s algorithm.
Execution Time	2	The system should be reactive and allow for fast planning and replanning for all of the cases.	The execution time value is the number of seconds of execution time required for the dynamic path planning system during implementation.
Number of Failures	5	The algorithm fails if it not able to plan any sub-path during the path planning task.	The failure score is obtained by using the frequency count of the total failures during the experiment.
Number of Collisions	4	The system should be robust to various scenarios and the number of collisions should be minimised to avoid failure of the system.	The score is the frequency count of the failures due to collisions during implementation.
Number of Deadlocks	4	A deadlock is a situation where two AMRs cannot continue following their pre-planned paths without causing a collision with each other.	The score is the frequency count of the failures due to deadlocks during implementation.

**Table 2:** Algorithm score comparison.

Criteria	Weight	Dĳkstra	RL with DDQN GRU–	Hybrid GA
			CNN Architecture	
Number of Failures	5	0	9	0
Number of Collisions	4	9	6	0
Number of Deadlocks	4	5	2	0
Weighted Path Length	3	1	1.09	1.01
Weighted Path Smoothness	3	1	2.64	0.95
Total Path Overlap	3	1	1.80	0.44
Execution Time (s)	2	0	1.29	0.78
Total		65.00	104.09	8.76

**Table 3:** Evaluation of the path quality results for Scenario 1.

Mobile Robot	Travel Distance (m)	Smoothness Score (rad)	Overlap Score (m)
Mobile Robot 1	86.5600 ± 3.1309	29.1226 ± 5.0598	24.4800 ± 4.5146
Mobile Robot 2	69.0400 ± 2.5632	26.7664 ± 5.4410	24.4800 ± 4.5146

**Table 4:** Evaluation of the path quality results for Scenario 2.

Factor	Travel Distance (m)	Smoothness Score (rad)	Overlap Score (m)
Mobile Robot 1	68.8600 ± 5.0242	24.8186 ± 4.6316	15.4000 ± 3.0305
Mobile Robot 2	70.8000 ± 2.8284	27.2690 ± 5.8106	15.4000 ± 3.0305

**Table 5:** Evaluation of the path quality results for Scenario 3.

Factor	Travel Distance (m)	Smoothness Score (rad)	Overlap Score (m)
Mobile Robot 1	84.8400 ± 2.2889	28.2920 ± 4.3829	17.5000 ± 2.6049
Mobile Robot 2	48.5200 ± 1.7052	16.6640 ± 3.1921	17.5000 ± 2.6049

**Table 6:** Evaluation of the path quality results for Scenario 4.

Factor	Travel Distance (m)	Smoothness Score (rad)	Overlap Score (m)
Mobile Robot 1	22.2400 ± 14.4682	6.2489 ± 3.2426	10.8400 ± 3.9865
Mobile Robot 2	65.0800 ± 6.9424	27.7032 ± 7.5232	10.8400 ± 3.9865

**Table 7:** Evaluation of the path quality results for Scenario 5.

Factor	Travel Distance (m)	Smoothness Score (rad)	Overlap Score (m)
Mobile Robot 1	75.8000 ± 19.3116	24.5004 ± 10.2933	14.5800 ± 5.1554
Mobile Robot 2	67.7000 ± 19.1857	28.2682 ± 9.4582	14.5800 ± 5.1554

**Table 8:** Evaluation of the path quality results for Scenario 6.

Factor	Travel Distance (m)	Smoothness Score (rad)	Overlap Score (m)
Mobile Robot 1	99.4800 ± 13.9023	44.7806 ± 7.4470	18.6200 ± 4.1151
Mobile Robot 2	69.8000 ± 3.6197	23.5491 ± 5.4803	18.6200 ± 4.1151

## Data Availability

The original contributions presented in this study are included in the article. Further inquiries can be directed to the corresponding author.

## Abbreviations

The following abbreviations are used in this manuscript:

ACO	Ant Colony Optimisation
AMR	Autonomous Mobile Robot
CBD	Cell-Based Decomposition
CNN	Convolutional Neural Network
DDQN	Double Deep Q-Network
FMS	Flexible Manufacturing System
FNN	Feedforward Neural Network
GA	Genetic Algorithm
GRU	Gated Recurrent Units
IEP	Iterative Exclusion Principle
ISO	International Organisation for Standardisation
MAS	Multi-Agent System
MPCA	Multi-Party Collision Avoidance
RL	Reinforcement Learning
PCA	Predictive Collision Avoidance
PP	Priority-based Planning
PSO	Particle Swarm Optimisation
